# Fauna Europaea: Gastrotricha

**DOI:** 10.3897/BDJ.3.e5800

**Published:** 2015-08-14

**Authors:** Maria Balsamo, Jean-Loup d`Hondt, Jacek Kisielewski, M. Antonio Todaro, Paolo Tongiorgi, Loretta Guidi, Paolo Grilli, Yde de Jong

**Affiliations:** ‡ Università di Urbino, Urbino, Italy; § Museum National d`Histoire Naturelle, Paris, France; | Adam Mickiewicz University, Poznań, Poland; ¶ Università di Modena e Reggio Emilia, Modena, Italy; # University of Amsterdam - Faculty of Science, Amsterdam, Netherlands; ¤ Museum für Naturkunde, Berlin, Germany

**Keywords:** Fauna Europaea, Biodiversity Informatics, Europe, Taxonomic indexing, Zoology, Biodiversity, Taxonomy, Gastrotricha, fresh waters

## Abstract

*Fauna Europaea* provides a public web-service with an index of scientific names (including important synonyms) of all living European land and freshwater animals, their geographical distribution at country level (up to the Urals, excluding the Caucasus region), and some additional information. The *Fauna Europaea* project covers about 230,000 taxonomic names, including 130,000 accepted species and 14,000 accepted subspecies, which is much more than the originally projected number of 100,000 species. This represents a huge effort by more than 400 contributing specialists throughout Europe and is a unique (standard) reference suitable for many users in science, government, industry, nature conservation and education.

Gastrotricha are a meiobenthic phylum composed of 813 species known so far (2 orders, 17 families) of free-living microinvertebrates commonly present and actively moving on and into sediments of aquatic ecosystems, 339 of which live in fresh and brackish waters. The Fauna Europaea database includes 214 species of Chaetonotida (4 families) plus a single species of Macrodasyida *incertae sedis*. This paper deals with the 224 European freshwater species known so far, 9 of which, all of Chaetonotida, have been described subsequently and will be included in the next database version. Basic information on their biology and ecology are summarized, and a list of selected, main references is given. As a general conclusion the gastrotrich fauna from Europe is the best known compared with that of other continents, but shows some important gaps of knowledge in Eastern and Southern regions.

## Introduction

The European Commission published the European Community Biodiversity Strategy, providing a framework for development of Community policies and instruments in order to comply with the Convention on Biological Diversity. This Strategy recognises the current incomplete state of knowledge at all levels concerning biodiversity, which is a constraint on the successful implementation of the Convention. Fauna Europaea contributes to this Strategy by supporting one of the main themes: to identify and catalogue the components of European biodiversity into a database in order to serve as a basic tool for science and conservation policies.

With regard to biodiversity in Europe, both science and policies depend on a knowledge of its components. The assessment of biodiversity, monitoring changes, sustainable exploitation of biodiversity, and much legislative work depend upon a validated overview of taxonomic biodiversity. Towards this end Fauna Europaea plays a major role, providing a web-based information infrastructure with an index of scientific names (including important synonyms) of all living European land and freshwater animals, their geographical distribution at country level and some additional useful information. In this sense, the Fauna Europaea database provides a unique reference for many user-groups such as scientists, governments, industries, conservation communities and educational programs.

Fauna Europaea started in 2000 as an EC-FP5 four-years project, delivering its first release in 2004 (Jong et al. 2014). After thirteen years of steady progress, in order to efficiently disseminate the Fauna Europaea results and to increase the acknowledgement of the Fauna Europaea contributors, novel e-Publishing tools have been applied to prepare data-papers of all major taxonomic groups. For this purpose a special Biodiversity Data Journal Series has been compiled, called Contributions on Fauna Europaea. This work was initiated during the ViBRANT project and is further supported by the recently started EU BON project. This paper holds the first publication of the Fauna Europaea Gastrotricha data sector as a BDJ data paper.

Within the EU BON project also further steps will be made to implement *Fauna Europaea* as a basic tool and standard reference for biodiversity research and to evaluate taxonomic expertise capacity in Europe. The *Fauna Europaea* data-papers will contribute to a quality assessement on biodiversity data by providing estimates on gaps in taxonomic information and knowledge.

## General description

### Purpose

The Fauna Europaea is a database of the scientific names and distribution of all living, currently known European land and fresh-water animal species assembled by a large network of experts, using advanced electronic tools for data collations and validation routines. An extended description of the Fauna Europaea project can be found in Jong et al. 2014). A summary is given in the sections below.

**Figure 1.**
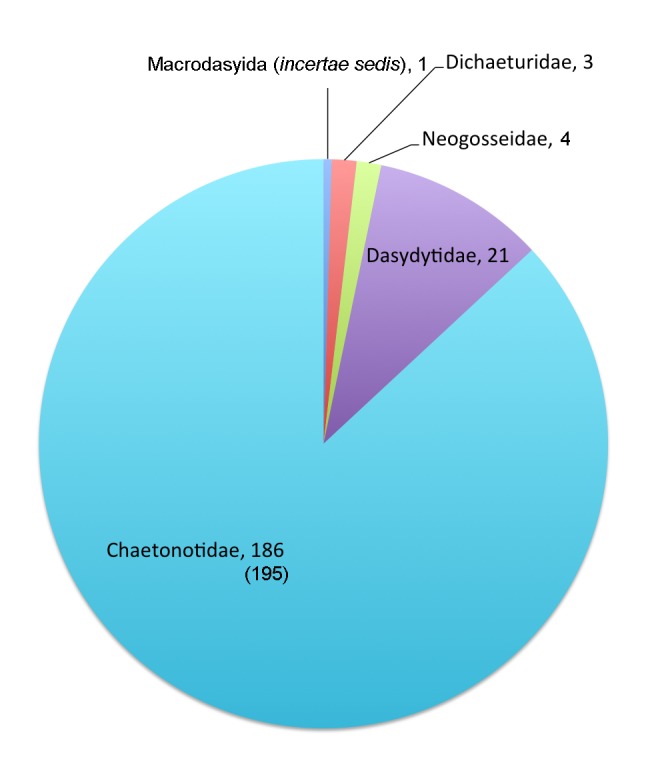
FaEu data set Gastrotricha species per family: the updated number of species is reported in brackets. See Table 1​ for family statistics.

The Gastrotricha are one of the 58 Fauna Europaea major taxonomic groups, and currently number 326 freshwater species worldwide, 224 of which reported from Europe. The data were acquired and checked by 5 specialists. No division of the taxa groups to be checked has been set among the specialists, thus all the specialists have to be considered responsible for the available data (Fig. 1​, Table 1).

### Additional information

**Gastrotricha**

The phylum Gastrotricha currently (May 2015) counts 813 species of free-living, microinvertebrates commonly present and actively moving on and into the sediments of aquatic ecosystems where they represent a significant component of meiofauna, with densities up to 364 ind/cm^2^ in marine sands and 168 ind/cm^2^ in freshwater sediments (Nesteruk 1993, Nesteruk 1996, Todaro and Hummon 2008).

Gastrotricha have ‘Aschelminthes’ or ‘pseudocoelomates’ features such as a worm-like body, a complete intestine, and a primary body cavity even if almost vestigial (Hyman 1951). Morphological and ultrastructural studies support a sister-group relationship of Gastrotricha with Cycloneuralia or with Ecdysozoa, whereas molecular analyses mainly carried out on the gene 18S rDNA have suggest to include the phylum into the Platyzoa, close to Gnathostomulida and acoelomate forms (for an overview of the major phylogenetic scenarios concerning Gastrotricha among the Bilateria as well as the internal relationships of the phylum, see Balsamo et al. 2010 and Kieneke and Schmidt-Rhaesa 2015). Recent studies based on a trascriptomic-phylogenomic approach found the gastrotrichs to be the sister taxon of the Platyhelminthes (e.g. Egger et al. 2015).

Classical morphological taxonomy recognizes two orders, Macrodasyida and Chaetonotida, quite different in general morphology, biology and ecology (Balsamo and Todaro 2002, Balsamo et al. 2014, Kieneke and Schmidt-Rhaesa 2015).

The order Macrodasyida includes 356 species (10 families, 35 genera), all interstitial, marine or brackish-water except for only 2 freshwater species. The marine monotypic genus *Hemidasys* has been considered extinct by Hummon and Todaro (2010). The vast majority of Chaetonotida, 335 species, colonize fresh waters, with a clear preference for eutrophic habitats, where most species known so far live as epiphytic or periphytic. Only about 70 freshwater species have been found in psammic habitats, and less than 35 species are known from sediments of running waters. Two entire families (Dasydytidae and Neogosseidae), with about 50 species, are semipelagic or fully planktonic: the colonization of water column corresponds to specific, characteristic morphological adaptations (Kisielewski 1981, Kisielewski 1990, Schwank 1990, Ricci and Balsamo 2000, Balsamo et al. 2008, Todaro et al. 2013, Kånneby and Todaro 2015).

Gastrotrichs share several morphological and physiological features with other groups of freshwater microinvertebrates: among them are small size, soft body, worm-like body shape, high contractility, adhesive glands, ciliary locomotion, triradiate pharynx with well-developed musculature, short life cycle, common parthenogenic reproduction, production of resting stages (d’Hondt 1971). But the adaptive potential of freshwater Gastrotricha is relatively limited compared to other animal phyla like Rotifera or Nematoda, so that their distribution appears to be narrower and to current knowledge it does not extend to extreme habitats.

The diversity of the phylum Gastrotricha is not very high, but these animals, as a part of the microphagous benthic community, play a significant ecological role in aquatic environments, linking the microbial loop to the higher trophic levels (Balsamo et al. 2014).

**Taxonomy and taxonomical issues**

The order Macrodasyida includes only 2 freshwater species: *Redudasys fornerise*, belonging to the family Redudasyidae (which also comprises a marine species, see Todaro et al. 2012), and *Marinellina flagellata*, which was previously assigned either to Macrodasyida (Ruttner-Kolisko 1955, Kisielewski 1987) or to Chaetonotida, in the freshwater family Dichaeturidae (Remane 1961) and is now considered a Macrodasyida *incertae sedis*. It is the only Macrodasyida species known from Europe, where it was found only once in Austria (Ruttner-Kolisko 1955).

The order Chaetonotida encompasses 335 freshwater species and 4 strictly brackish-water species. Four families (12 genera) are exclusively freshwater (Dasydytidae, Neogosseidae, Proichthydiidae, Dichaeturidae), and the largest family, Chaetonotidae, includes both marine and freshwater species (2 genera exclusively marine, 5 freshwater and 5 including marine, brackish-water and freshwater species).

Thanks to recent studies, the current systematics is relatively stable for the order Macrodasyida (Todaro et al. 2006, Hummon and Todaro 2010, Todaro et al. 2011, Todaro et al. 2012, Guidi et al. 2014, Todaro et al. 2014), except for the monotypic genus *Marinellina* (still *incertae sedis*). By contrast, the taxonomy of the order Chaetonotida has been repeatedly revised in the last decades and is still unstable especially at the species level (Schwank 1990, Kisielewski 1991, Kisielewski 1997, Kisielewski 1998, Leasi and Todaro 2008, Balsamo et al. 2009, Hummon and Todaro 2010, Balsamo et al. 2014). Furthermore, a phylogenetic study based on molecular markers found the largest chaetonotidan family, Chaetonotidae, and most of the genera included in it to be non-monophyletic (Kånneby et al. 2013).

Actually, the taxonomy of the phylum has been founded on morphological traits both of the external structure and the internal anatomy, like the shape and size of the cuticular elements, the organization of the reproductive system and the fine structure of spermatozoa (Marotta et al. 2005, Hummon and Todaro 2010, Guidi et al. 2014). However, especially in Chaetonotida, several of these taxonomical characters appear to vary, even considerably, at species level and in some cases also at genus level, which makes taxonomic identification quite problematic (Schwank 1990, Balsamo et al. 2009).

It must be said that Gastrotricha, small and diaphanous, should be observed alive with a very good microscopical equipment in order to recognize and measure all the morphological details taxonomically important (Balsamo et al. 2014). That is not always possible, considering the technical problems of collecting samples, maintaining them in the lab, searching for in them in the sediments under a stereomicroscope, and then isolating single specimens be anesthetized in some manner and to allow proper observation. However, several freshwater species can be suitably fixed and observed later with good results (Giere 2009).

The recent introduction of molecular analyses to Gastrotricha has opened new perspectives in the study of phylogenetical relationships; an integrative taxonomical approach using both morphological and molecular methods seems essential to effectively revise the current classification according to phylogenetic relationships (Kånneby et al. 2012, Paps and Riutort 2012, Todaro et al. 2012, Kånneby et al. 2013, Todaro et al. 2014).

We may expect a future increase in species numbers of Chaetonotidae and possibly of Dasydytidae. A reliable assessment of the estimated species number cannot be advanced due to the sporadic nature of faunistic findings and samplings, but a reasonable minimal estimate of possible increase in number of species known is advanced based on the current knowledge of unpublished data (Table 1).

**European freshwater species**

The Fauna Europaea database (version 2.4, January 2011) includes 215 species, 214 of which belong to the order Chaetonotida (4 families) and a single species *incertae sedis* is a member of the order Macrodasyida.

Eight additional species of Chaetonotida have been described and another one has been recorded first for Europe after the release of the database, so that currently European freshwater species of Gastrotricha known so far add up to 224 overall (223 Chaetonotida + 1 Macrodasyida), that are considered in the present paper.

A synthesis of European taxa is presented below.

**Figure 2.**
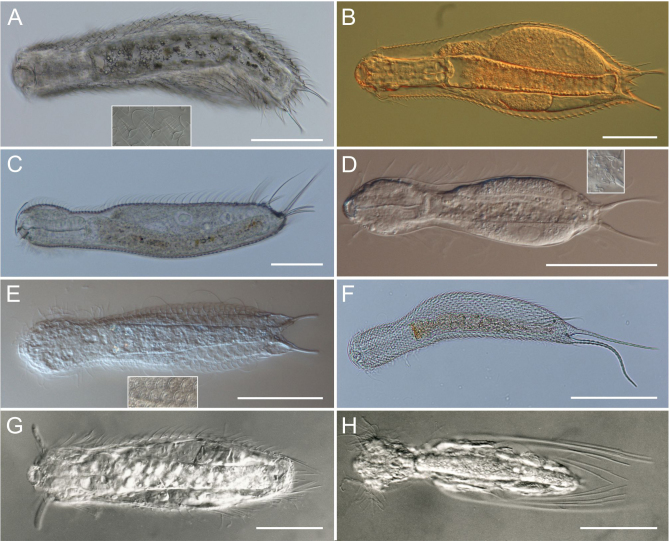
Common and rare representatives of European freshwater gastrotrichs. **A-F**, Fam. Chaetonotidae. **A**, Chaetonotus (Captochaetus) robustus (insert shows the peculiar scales); **B**, Chaetonotus (Chaetonotus) polyspinosus; **C**, Chaetonotus (Lepidochaetus) zelinkai; **D**, *Ichthydium skandicum* (insert shows the scales of the furcal base); **E**, *Lepidodermella squamata* (insert shows the dorsal scales); **F**, *Polymerurus nodicaudus*; *G*, Fam. *Neogosseidae*, *Neogossea antennigera*; **H**, Fam. Dasydytidae, *Stylochaeta fusiformis*. Scale bars: A, F =100 µm; B-E, G, H = 50 µm. Original images can be found here: Suppl. materials 2, 3, 4, 5, 6, 7, 8, 9.

**Family Chaetonotidae (Fig. 2 A-F)**

Tenpin-like body, 84-770 μm in length.Two adhesive tubes forming the caudal ‘furca’. Four cephalic ciliary tufts. Cuticle generally provided with ornamentations of various shape and size, in some cases smooth. Two longitudinal ventral ciliary locomotory bands. Pharynx generally cylindrical, slightly widened posteriorly. Parthenogenic; rudimentary testes or aberrant spermatozoa recorded in several species.

This family is the largest one of the order, and includes most of the epibenthic and periphytic species colonizing standing waters as well as the few interstitial species known from psammic habitats.

At global scale the family Chaetonotidae includes 277 freshwater species (10 genera).

In European fresh waters the family is currently represented by 195 species in 8 genera: 5 genera include also marine species *Aspidiophorus* (15 species), *Chaetonotus* (125), *Heterolepidoderma* (18), *Ichthydium* (19), *Lepidodermella* (7), whereas 3 genera are exclusively freshwater: *Fluxiderma* (3), *Polymerurus* (7), *Rhomballichthys* (1).

These numbers include 8 new species and a single new record for Europe that have been reported after the release of the Fauna Europaea database in January 2011.

Newly described species

Chaetonotus (Chaetonotus) eximius Kolicka, Kisielewski, Nesteruk & Zawierucha, 2013

Chaetonotus (Chaetonotus) pravus Kolicka, Kisielewski, Nesteruk & Zawierucha, 2013

Chaetonotus (Primochaetus) veronicae Kånneby, 2013

Chaetonotus (Tristratachaetus) rhombosquamatus Kolicka, Kisielewski, Nesteruk & Zawierucha, 2013

*Heterolepidoderma acidophilum* Kånneby, Todaro & Jondelius, 2012

*Heterolepidoderma joermungandri* Kånneby, 2011

*Heterolepidoderma trapezoidum* Kånneby, 2011

*Lepidodermella intermedia* Kånneby, Todaro & Jondelius, 2012

New species record for Europe

Chaetonotus (Primochaetus) soberanus Grosso & Drahg, 1983

**Family Dasydytidae (Fig. 2 H)**

Tenpin-like or bottle-shaped body, 75-291 μm in length (caudal spines excluded). Caudal furca absent: rounded or truncated caudal end with 2 lobes bearing bristles or 2 to few long spines. Two cephalic ciliary tufts and a transversal band extending from ventral to dorsal side. Cuticle smooth, rarely with few faint scales. Ventrolateral spines short to very long, simple or more often barbed, isolated or in groups, inserted on the trunk region and sometimes movable. Few oblique ciliary locomotory series or tufts, rarely 2 bands (*Haltidytes*). Pharynx cylindrical with1-2 bulbs. Parthenogenic; rudimentary testes or aberrant spermatozoa recorded in several species.

All species of Dasydytidae are freshwater, semiplanktonic and planktonic in standing waters.

At global scale the family Dasydytidae includes 42 species in 7 genera.

In European fresh waters the family Dasydytidae is represented by 21 species (6 genera): *Anacanthoderma* (2), *Chitonodytes* (3), *Dasydytes* (3), *Haltidytes* (3), *Setopus* (6) and *Stylochaeta* (4).

**Family Dichaeturidae**

Cylindrical body, 98-150 μm in length. Two pairs of adhesive tubes forming the caudal furca. Cephalic ciliature uniform and continuous with 2 ventral ciliary locomotory bands. Cuticle smooth. A dorsal, transverse series of some thin, straight bristles or spines anterior to the furca. Pharynx cylindrical. Sexuality unknown.

All species of family Dichaeturidae are freshwater, very rare, semiplanktonic in standing waters.

At global scale the family Dichaeturidae includes 4 species of a single genus.

In European fresh waters the family Dichaeturidae is represented by 3 species in the single genus *Dichaetura*.

**Family Neogosseidae (Fig. 2 G)**

Tenpin-like body, 90-310 μm in length (caudal spines excluded). Caudal furca absent: trunk end rounded or truncated with 2 short, caudal protuberances and 2 pairs or an unpaired median group of long simple or barbed spines. One dorsal and 2 ventral cephalic interrupted transverse ciliary bands. Cuticle smooth or with numerous fine spined scales. Several pairs of ventral ciliary locomotory tufts or oblique bands. Thick pharynx with 1-4 bulbs. Parthenogenic.

All the species of family Neogosseidae are freshwater, semiplanktonic or planktonic in standing waters.

At global scale the family Neogosseidae is composed of 9 species in 2 genera.

In European fresh waters the family is represented by 4 species in 2 genera: *Neogossea* (3) and *Kijanebalola* (1 species, finding doubtful).

**Family Proichthydiidae**, the only other family of Chaetonotida exclusively freshwater, includes 2 species of 2 monotypic genera *(Proichthydium, Proichthydioides*): it is known from Brazil and Japan but has not yet been reported from Europe.

**Order Macrodasyida**

**Family *incertae sedis***

*Marinellina flagellata* Ruttner-Kolisko, 1955

Cylindrical body, 220 μm in length. Two pairs of adhesive tubes forming the caudal furca. Two cephalic ventrolateral adhesive tubes. Numerous long sensory bristles on the head and along the body. Cuticle smooth. Ventral locomotory ciliature undescribed. Thick, cylindrical pharynx. Sexuality unknown.

This single species of the genus was found only once from Austrian interstitial fresh waters.

## Project description

### Title

This BDJ data paper includes the taxonomic indexing efforts in the Fauna Europaea on European Gastrotricha covering the first two versions of Fauna Europaea worked on between 2000 and 2013 (up to version 2.6).

### Personnel

The taxonomic framework of Fauna Europaea includes partner institutes, providing taxonomic expertise and information, and expert networks taking care about data collation.

Every taxonomic group is covered by at least one Group Coordinator responsible for the supervision and integrated input of taxonomic and distributional data of a particular group. The Fauna Europaea checklist would not have reached its current level of completion without the input from several groups of specialists. The formal responsibility of collating and delivering the data of relevant families rested with a number of Taxonomic Specialists (see Table 1).

For Gastrotricha the responsible Group Coordinator and Taxonomic specialist is Maria Balsamo, who is also Taxonomic Specialist, together with the other Associated Specialists listed in Table 2. A more detail overview of the Fauna Europaea classification and expertise network for Gastrotricha can be found here: http://www.faunaeur.org/experts.php?id=21.

Data management tasks are taken care about by the Fauna Europaea project bureau. During the project phase (until 2004) a network of principal partners took responsability for various management tasks: Zoological Museum Amsterdam (general management & system development), Zoological Museum of Copenhagen (data collation), National Museum of Natural History in Paris (data validation) and Museum and Institute of Zoology in Warsaw (NAS extension). After the formal project ending (2004 till 2014) all tasks have been undertaken by the Zoological Museum Amsterdam. Since 2013 the data servers are hosted at the Museum für Naturkunde in Berlin (migrated from ZMA-UvA).

On the available expert capacity, presently, in Europe faunistic, systematic and taxonomical studies on freshwater Gastrotricha species are actively carried out in Italy (University of Urbino), in France (MNHN) and in Sweden (NRM) by around five specialists. Some additional ultrastructural and phylogenetical work is done on Gastrotricha in Germany (Senckenberg &  University of Hamburg). Outside Europe around six more specialists contribute to the taxonomy of (marine and freshwater) Gastrotricha.

### Study area description

The area study covers the European mainland (Western Palearctic), including the Macaronesian islands, excluding the Caucasus, Turkey, Arabian Peninsula and Northern Africa (see: Geographic coverage).

### Design description

*Standards*. Group coordinators and taxonomic specialists have to deliver the (sub)species names according to strict standards. The names provided by Fauna Europaea are scientific names. The taxonomic scope includes issues like, (1) the definition of criteria used to identify the accepted species-group taxa, (2) the hierarchy (classification scheme) for the accommodation of the all accepted species and (3), relevant synonyms, and (4) the correct nomenclature. The Fauna Europaea 'Guidelines for Group Coordinators and Taxonomic Specialists', include the standards, protocols, scope, and limits that provide the instructions for all more than 400 specialists contributing to the project, strictly following the provisions of the current edition of the International Code of Zoological Nomenclature.

**Figure 3.**
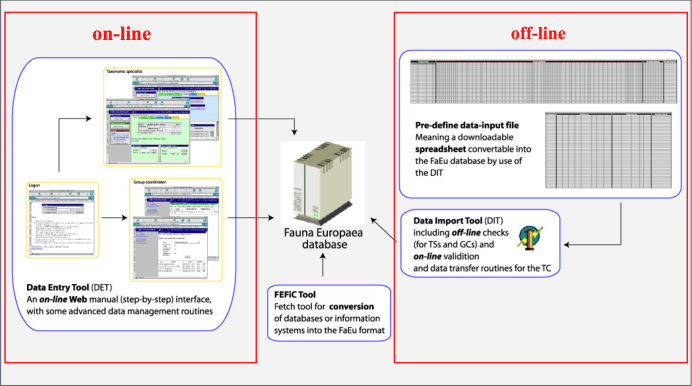
Fauna Europaea on-line (browser interfaces) and off-line (spreadsheets) data entry tools.

*Data management*. The data records could either be entered offline into a preformatted MS-Excel worksheet or directly into the Fauna Europaea transaction database using an online browser interface (Fig. 3).

*Data set*. The Fauna Europaea basic data set consists of: accepted (sub)species names (including authorship), synonym names (including authorship), a taxonomic hierarchy/classification, misapplied names (including misspellings and alternative taxonomic views), homonym annotations, expert details, European distribution (at country level), Global distribution (only for European species), taxonomic reference (optional), occurrence reference (optional).

### Funding

*Fauna Europaea* was funded by the European Commission under the Fifth Framework Programme and contributed to the Support for Research Infrastructures work programme with Thematic Priority Biodiversity (EVR1-1999-20001) for a period of four years (1 March 2000 - 1 March 2004), including a short 'NAS extension', allowing EU candidate accession countries to participate. Follow-up support was given by the EC-FP6 EDIT project (GCE 018340), by the EC-FP7 PESI project (RI-223806) and by the EC-FP7 ViBRANT project (RI-261532). Continuing management and hosting of the Fauna Europaea services was supported by the University of Amsterdam (Zoological Museum Amsterdam) and SARA/Vancis. Recently the hosting of Fauna Europaea was taken over by the Museum für Naturkunde in Berlin, supported by the EC-FP7 EU BON project (grant agreement №308454).

For preparing the Gastrotricha data set additional support was received from MIUR (Italian Ministry of University and of Scientific and Technological Research).

## Sampling methods

### Study extent

See spatial coverage and geographic coverage descriptions.

### Sampling description

Fauna Europaea data have been assembled by principal taxonomic experts, based on their individual expertise, including literature sources, collection research, and field observations. In total no less than 476 experts contributed taxonomic and/or faunistic information for Fauna Europaea. The vast majority of the experts are from Europe (including EU non-member states). As a unique feature, Fauna Europaea funds were set aside for rewarding/compensating for the work of taxonomic specialists and group coordinators.

To facilitate data transfer and data import, sophisticated on-line (web interfaces) and off-line (spreadsheets) data-entry routines were built, integrated within an underlying central Fauna Europaea transaction database (see Fig. 3) This includes advanced batch data import routines and utilities to display and monitor the data processing within the system. In retrospect, it seems that the off-line submission of data was probably the best for bulk import during the project phase, while the on-line tool was preferred to enter modifications in later versions. This system works well, but will be replaced after 2013.

A first release of the Fauna Europaea index via the web-portal has been presented at 27^th^ of September 2004, the most recent release (version 2.6.2) was launched at 29 August 2013. An overview of Fauna Europaea releases can be found here: http://www.faunaeur.org/about_fauna_versions.php.

### Quality control

Fauna Europaea data are unique in a sense that they are fully expert based. Selecting leading experts for all groups assured the systematic reliability and consistency of the Fauna Europaea data. Furthermore, all Fauna Europaea data sets are intensively reviewed at regional and thematic validation meetings, at review sessions on taxonomic symposia (for some groups), by Fauna Europaea Focal Points (during the FaEu-NAS and PESI projects) and by various end-users sending annotations using the web form at the web-portal. Additional validation on gaps and correct spelling was effected at the validation office in Paris.

In general we expect to get taxonomic data for 99.3% of the known European fauna after the initial release. The faunistic coverage is not quite as good, but is nevertheless 90-95% of the total fauna. For Gastrotricha the current taxonomic coverage is about 96% (see Table 1), and the distribution of faunistic data by country is quite heterogeneous, according to the nationality of researchers.

Checks on technical and logical correctness of the data have been implemented in the data entry tools, including around 50 "Taxonomic Integrity Rules". This validation tool proved to be of huge value for both the experts and project management, and contributed significantly to preparation of a remarkably clean and consistent data set. This thorough reviewing makes Fauna Europaea the most scrutinised data sets in its domain.

The only other existing database specifically dedicated to freshwater species on a worldwide scale has been produced within FADA (Freshwater Animal Diversity Assessment Project), but it has not yet been published. A number of freshwater species appear also in the WoRMS (World Register of Marine Species) taxonomic database of Gastrotricha, since some species have been occasionally reported from brackish waters.

### Step description

By evaluating team structure and life cycle procedures (data-entry, validation, updating, etc.), clear definitions of roles of users and user-groups, according to the taxonomic framework were established, including ownership and read and writes privileges, and their changes during the project life-cycle. In addition, guidelines on common data exchange formats and codes have been issued (see also the 'Guidelines for Experts' document).

## Geographic coverage

### Description

**Figure 4.**
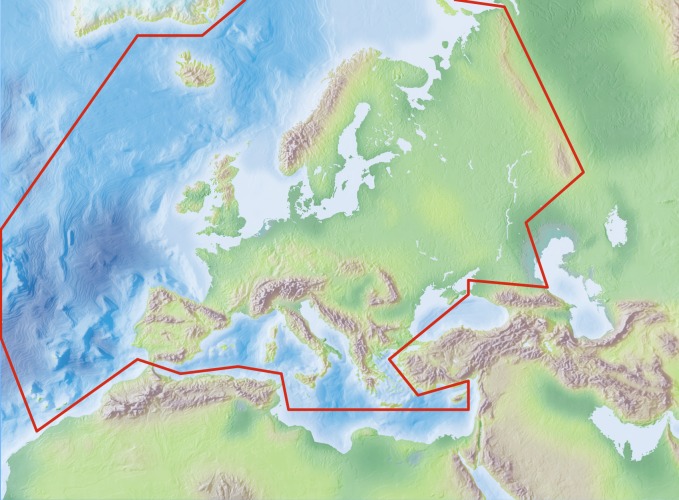
Fauna Europaea geographic coverage ('minimal Europe').

Species and subspecies distributions in Fauna Europaea are registered at least a country level, i.e. for political countries. For this purpose the FaEu geographical system basically follows the TDWG standards. The covered area includes the European mainland (Western Palearctic), plus the Macaronesian islands (excl. Cape Verde Islands), Cyprus, Franz Josef Land and Novaya Zemlya. Western Kazakhstan and the Caucasus are excluded (see Fig. 4).

The focus is on species (or subspecies) of European animals of terrestrial and freshwater environments. Species in brackish waters, occupying the marine/freshwater or marine/terrestrial transition zones, are generally excluded.

The four species of Chaetonotida only known from brackish waters have been included in the database, in which their particular habitat has been specified.

### Coordinates

Mediterranean (N 35°) and Arctic Islands (N 82°) Latitude; Atlantic Ocean (Mid-Atlantic Ridge) (W 30°) and Ural (E 60°) Longitude.

## Taxonomic coverage

### Description

This data paper covers the Gastrotricha content of Fauna Europaea, including 4 families, 214 species, 5 subspecies and 199 species synonyms of Chaetonotida and one species *incertae sedis* of Macrodasyida (Fig. 1, Table 1).

Not all the species described to date are included in the current version of the Fauna Europaea database. The next version of the Fauna Europaea database will be updated with the most recent records.

The placement of the genus *Marinellina*, the only one freshwater European Macrodasyida, is uncertain. In the database it was considered as a member of the family Turbanellidae in the order Macrodasyida Remane, 1925 (Rao and Clausen 1970), but since the taxonomic position of this genus is now actively debated, it is here reported as *'incertae sedis'* according to the recent literature.

### Taxa included

**Table:** 

Rank	Scientific Name	Common Name
kingdom	Animalia	
subkingdom	Eumetazoa	
phylum	Gastrotricha Metschnikoff, 1865	
order	Chaetonotida Remane, 1925 [Rao and Clausen,1970]	
family	Chaetonotidae Gosse, 1864	
genus	*Aspidiophorus* Voigt, 1903	
genus	*Chaetonotus* Ehrenberg, 1830	
subgenus	*Chaetonotus* (*Captochaetus*) Kisielewski, 1997	
subgenus	*Chaetonotus* (*Chaetonotus*) Ehrenberg, 1830	
subgenus	Chaetonotus (Lepidochaetus) Kisielewski, 1991	
subgenus	Chaetonotus (Primochaetus) Kisielewski 1997	
subgenus	Chaetonotus (Schizochaetonotus) Schwank, 1990	
subgenus	Chaetonotus (Tristratachaetus) Kolicka, Kisielewski, Nesteruk & Zawierucha, 2013	
subgenus	Chaetonotus (Wolterecka) Mola, 1932	
subgenus	Chaetonotus (Zonochaeta) Remane, 1927	
genus	*Fluxiderma* d'Hondt, 1974	
genus	*Heterolepidoderma* Remane, 1927	
genus	*Ichthydium* Ehrenberg, 1830	
genus	*Lepidodermella* Blake, 1933	
genus	*Polymerurus* Remane, 1927	
genus	*Rhomballichthys* Schwank, 1990	
family	Dasydytidae Daday, 1905	
genus	*Anacanthoderma* Marcolongo, 1910	
genus	*Chitonodytes* Remane, 1936	
genus	*Dasydytes* Gosse, 1851	
genus	*Haltidytes* Remane, 1936	
genus	*Setopus* Grünspan, 1908	
genus	*Stylochaeta* Hlava, 1904	
family	Dichaeturidae Remane, 1927	
genus	*Dichaetura* Lauterborn, 1913	
family	Neogosseidae Remane, 1929	
genus	*Kijanebalola* de Beauchamp, 1932	
genus	*Neogossea* Remane, 1927	

## Temporal coverage

**Living time period:** Currently living.

### Notes

Currently living animals in stable populations, largely excluding (1) rare/irregular immigrants, intruder or invader species, (2) accidental or deliberate releases of exotic (pet) species, (3) domesticated animals, (4) foreign species imported and released for bio-control or (5) foreign species largely confined to hothouses.

## Usage rights

### Use license

Open Data Commons Attribution License

### IP rights notes

*Fauna Europaea* data are licensed under CC BY SA version 4.0. The property rights of experts over their data is covered by their Fauna Europaea contract agreements. For more IPR details see: http://www.faunaeur.org/copyright.php.

## Data resources

### Data package title

Fauna Europaea - Gastrotricha

### Resource link

http://www.faunaeur.org/Data_papers/FaEu_Gastrotricha_2.6.2.zip

### Alternative identifiers

http://www.faunaeur.org/full_results.php?id=11241

### Number of data sets

2

### Data set 1.

#### Data set name

Fauna Europaea - Gastrotricha version 2.6.2 - species

#### Data format

CSV

#### Number of columns

25

#### Character set

UTF-8

#### Download URL

http://www.faunaeur.org/Data_papers/FaEu_Gastrotricha_2.6.2.zip

#### Description

**Data set 1.** 

Column label	Column description
datasetName	The name identifying the data set from which the record was derived (http://rs.tdwg.org/dwc/terms/datasetName).
version	Release version of data set.
versionIssued	Issue data of data set version.
rights	Information about rights held in and over the resource (http://purl.org/dc/terms/rights).
rightsHolder	A person or organization owning or managing rights over the resource (http://purl.org/dc/terms/rightsHolder).
accessRights	Information about who can access the resource or an indication of its security status (http://purl.org/dc/terms/accessRights).
taxonID	An identifier for the set of taxon information (http://rs.tdwg.org/dwc/terms/taxonID)
parentNameUsageID	An identifier for the name usage of the direct parent taxon (in a classification) of the most specific element of the scientificName (http://rs.tdwg.org/dwc/terms/parentNameUsageID).
scientificName	The full scientific name, with authorship and date information if known (http://rs.tdwg.org/dwc/terms/scientificName).
acceptedNameUsage	The full name, with authorship and date information if known, of the currently valid (zoological) taxon (http://rs.tdwg.org/dwc/terms/acceptedNameUsage).
originalNameUsage	The original combination (genus and species group names), as firstly established under the rules of the associated nomenclaturalCode (http://rs.tdwg.org/dwc/terms/originalNameUsage).
family	The full scientific name of the family in which the taxon is classified (http://rs.tdwg.org/dwc/terms/family).
familyNameId	An identifier for the family name.
genus	The full scientific name of the genus in which the taxon is classified (http://rs.tdwg.org/dwc/terms/genus).
subgenus	The full scientific name of the subgenus in which the taxon is classified. Values include the genus to avoid homonym confusion (http://rs.tdwg.org/dwc/terms/subgenus).
specificEpithet	The name of the first or species epithet of the scientificName (http://rs.tdwg.org/dwc/terms/specificEpithet).
infraspecificEpithet	The name of the lowest or terminal infraspecific epithet of the scientificName, excluding any rank designation (http://rs.tdwg.org/dwc/terms/infraspecificEpithet).
taxonRank	The taxonomic rank of the most specific name in the scientificName (http://rs.tdwg.org/dwc/terms/infraspecificEpithet).
scientificNameAuthorship	The authorship information for the scientificName formatted according to the conventions of the applicable nomenclaturalCode (http://rs.tdwg.org/dwc/terms/scientificNameAuthorship).
authorName	Author name information
namePublishedInYear	The four-digit year in which the scientificName was published (http://rs.tdwg.org/dwc/terms/namePublishedInYear).
Brackets	Annotation if authorship should be put between parentheses.
nomenclaturalCode	The nomenclatural code under which the scientificName is constructed (http://rs.tdwg.org/dwc/terms/nomenclaturalCode).
taxonomicStatus	The status of the use of the scientificName as a label for a taxon (http://rs.tdwg.org/dwc/terms/taxonomicStatus).
resourceDescription	An account of the resource, including a data-paper DOI (http://purl.org/dc/terms/description)

### Data set 2.

#### Data set name

Fauna Europaea - Gastrotricha version 2.6.2 - hierarchy

#### Data format

CSV

#### Number of columns

12

#### Character set

UTF-8

#### Download URL

http://www.faunaeur.org/Data_papers/FaEu_Gastrotricha_2.6.2.zip

#### Description

**Data set 2.** 

Column label	Column description
datasetName	The name identifying the data set from which the record was derived (http://rs.tdwg.org/dwc/terms/datasetName).
version	Release version of data set.
versionIssued	Issue data of data set version.
rights	Information about rights held in and over the resource (http://purl.org/dc/terms/rights).
rightsHolder	A person or organization owning or managing rights over the resource (http://purl.org/dc/terms/rightsHolder).
accessRights	Information about who can access the resource or an indication of its security status (http://purl.org/dc/terms/accessRights).
taxonName	The full scientific name of the higher-level taxon
scientificNameAuthorship	The authorship information for the scientificName formatted according to the conventions of the applicable nomenclaturalCode (http://rs.tdwg.org/dwc/terms/scientificNameAuthorship).
taxonRank	The taxonomic rank of the most specific name in the scientificName (http://rs.tdwg.org/dwc/terms/infraspecificEpithet).
taxonID	An identifier for the set of taxon information (http://rs.tdwg.org/dwc/terms/taxonID)
parentNameUsageID	An identifier for the name usage of the direct parent taxon (in a classification) of the most specific element of the scientificName (http://rs.tdwg.org/dwc/terms/parentNameUsageID).
resourceDescription	An account of the resource, including a data-paper DOI (http://purl.org/dc/terms/description)

## Supplementary Material

Supplementary material 1 Fauna Europaea Gastrotricha species version 3 (draft May 2015) Data type: xlsx
File: oo_45068.xlsx Maria Balsamo

Supplementary material 2 Figure 1A: Chaetonotus (Captochaetus) robustus (insert shows the peculiar scales); Scale bar =100 µm. Data type: jpg
Brief description: Original image of Figure 1A.
File: oo_48637.jpg Maria Balsamo

Supplementary material 3 Figure 1B: Fam. Chaetonotidae, Chaetonotus (Chaetonotus) polyspinosus; Scale bar =100 µm. Data type: jpg
Brief description: Original image of Figure 1B.
File: oo_48638.jpg Maria Balsamo

Supplementary material 4 Figure 1C: Fam. Chaetonotidae, Chaetonotus (Lepidochaetus) zelinkai; Scale bar =100 µm. Data type: jpg
Brief description: Original image of Figure 1C.
File: oo_48639.jpg Maria Balsamo

Supplementary material 5 Figure 1D: Fam. Chaetonotidae, Ichthydium skandicum (insert shows the scales of the furcal base); Scale bar =100 µm. Data type: jpg
Brief description: Original image of Figure 1D.
File: oo_48640.jpg Maria Balsamo

Supplementary material 6 Figure 1E: Fam. Chaetonotidae, Lepidodermella squamata (insert shows the dorsal scales); Scale bar =100 µm. Data type: jpg
Brief description: Original image of Figure 1E.
File: oo_48641.jpg Maria Balsamo

Supplementary material 7 Figure 1F: Fam. Chaetonotidae, Polymerurus nodicaudus; Scale bar =100 µm. Data type: jpg
Brief description: Original image of Figure 1F.
File: oo_48642.jpg Maria Balsamo

Supplementary material 8 Figure 1G: Fam. Neogosseidae, Neogossea antennigera; Scale bar = 50 µm. Data type: jpg
Brief description: Original image of Figure 1G.
File: oo_48643.jpg Maria Balsamo

Supplementary material 9 Figure 1H: Fam. Dasydytidae, Stylochaeta fusiformis; Scale bar = 50 µm. Data type: jpg
Brief description: Detailed image of composted Figure 1.
File: oo_48644.jpg Maria Balsamo

## Figures and Tables

**Table 1.** Responsible specialists per family in Gastrotricha. The actual number of databased species, the total number of known/described species (showing a potential information gap) plus an estimate of the total number of existing species (described plus undescribed) (showing a potential knowledge gap) for Europe are given per family (see also Fig. 1).

**TAXONOMY**		**EUROPE**			
**FAMILY**	**SPECIALIST(S)**	**DATABASED SPECIES (Fauna Europaea v2)**	**TOTAL DESCRIBED SPECIES**	**TOTAL ESTIMATED SPECIES**	**COMMENT**
Chaetonotidae	Maria Balsamo; J. Kisielewski; J.L. d'Hondt; P. Tongiorgi; M.A.Todaro	186	195	≥ 300	
Dasydytidae	Maria Balsamo; J. Kisielewski; J.L. d'Hondt; P. Tongiorgi; M.A.Todaro	21	21	≥ 30	
Dichaeturidae	Maria Balsamo; J. Kisielewski; J.L. d'Hondt; P. Tongiorgi; M.A.Todaro	3	3	≥ 5	
Neogosseidae	Maria Balsamo; J. Kisielewski; J.L. d'Hondt; P. Tongiorgi; M.A.Todaro	4	4	≥ 10	Here *Neogossea fasciculata beauchampi* is treated as a species, being the only subspecies present in Europe
*incertae sedis* (Macrodasyida)	Maria Balsamo; J. Kisielewski; J.L. d'Hondt; P. Tongiorgi; M.A.Todaro	1	1	?	Placement of *Marinellina* under consideration

**Table 2.** Responsible associated specialists in Gastrotricha.

**GROUP or AREA**	**SPECIALIST(S)**	**Version**
Chaetonotida	Jacek Kisielewski	1 & 2
Chaetonotida	Jean-Loup d'Hondt	1 & 2
Chaetonotida	Paolo Tongiorgi	1 & 2 (now retired)
Chaetonotida	Antonio Todaro	1 & 2 (now working on marine species)
Chaetonotida	Tobias Kånneby	new
Chaetonotida	Malgorzata Kolicka	new
Chaetonotida	Paolo Grilli	new
Chaetonotida	Loretta Guidi	new
Chaetonotida	Alexander Kieneke	new
