# Supplementary figures and images for: Fauna Europaea: Gastrotricha
Source: Biodivers Data J. 2015 Aug 14;(3):e5800. doi: 10.3897/BDJ.3.e5800 (PMC4563153; doi:10.3897/BDJ.3.e5800)

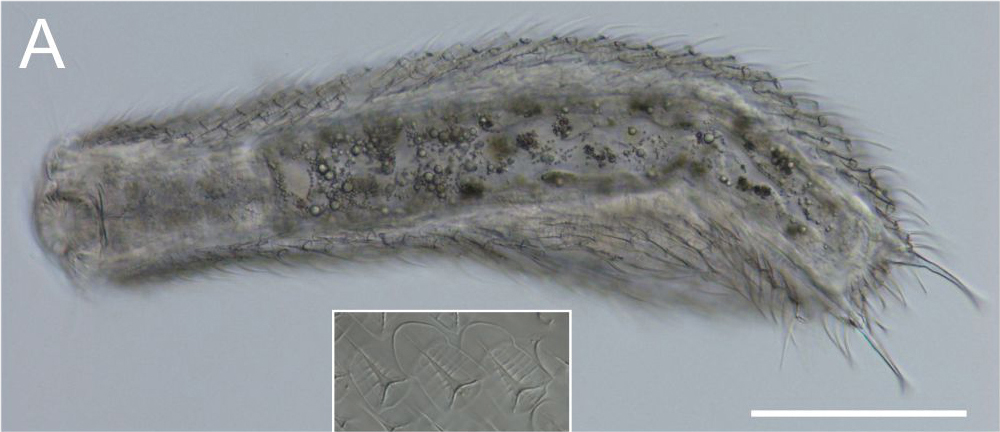

Supplement: Supplementary material 2 — Figure 1A: Chaetonotus (Captochaetus) robustus (insert shows the peculiar scales); Scale bar =100 µm. [file biodiversity_data_journal-3-e5800-s002.jpg]

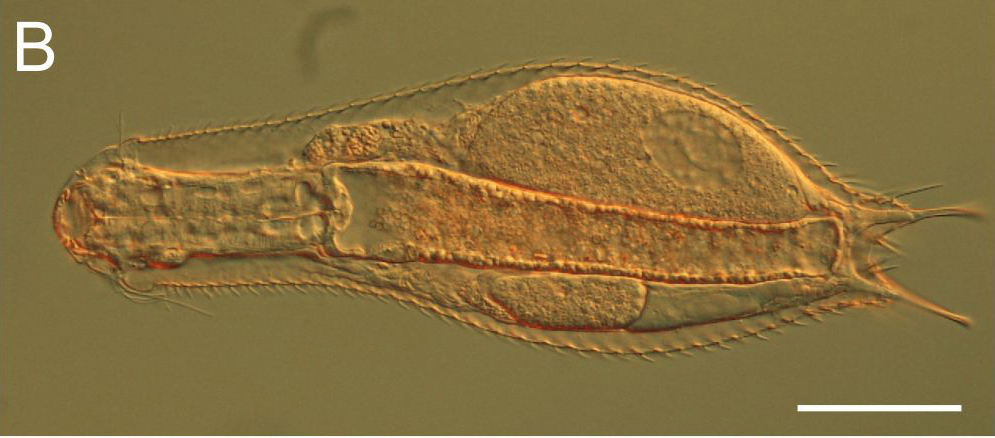

Supplement: Supplementary material 3 — Figure 1B: Fam. Chaetonotidae, Chaetonotus (Chaetonotus) polyspinosus; Scale bar =100 µm. [file biodiversity_data_journal-3-e5800-s003.jpg]

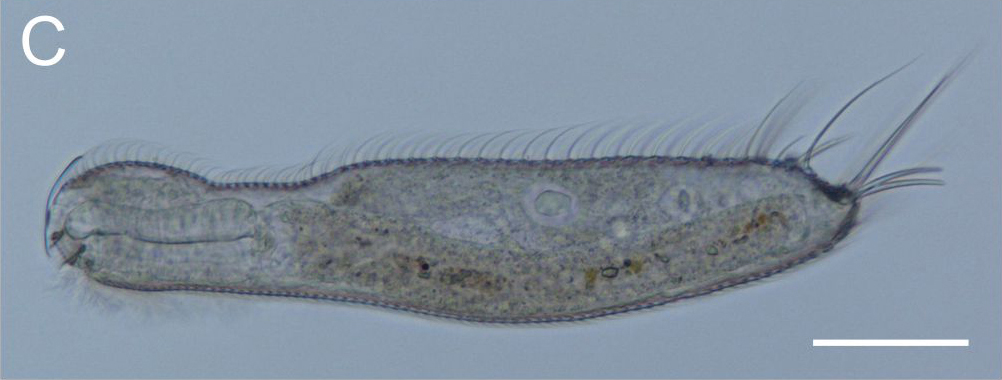

Supplement: Supplementary material 4 — Figure 1C: Fam. Chaetonotidae, Chaetonotus (Lepidochaetus) zelinkai; Scale bar =100 µm. [file biodiversity_data_journal-3-e5800-s004.jpg]

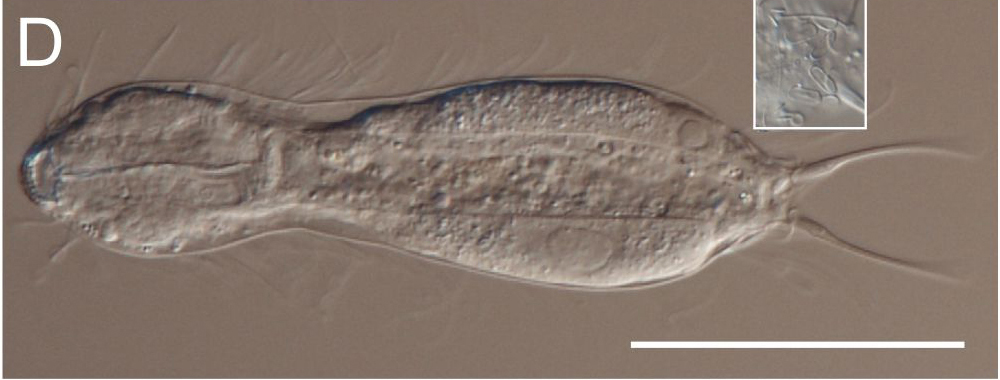

Supplement: Supplementary material 5 — Figure 1D: Fam. Chaetonotidae, Ichthydium skandicum (insert shows the scales of the furcal base); Scale bar =100 µm. [file biodiversity_data_journal-3-e5800-s005.jpg]

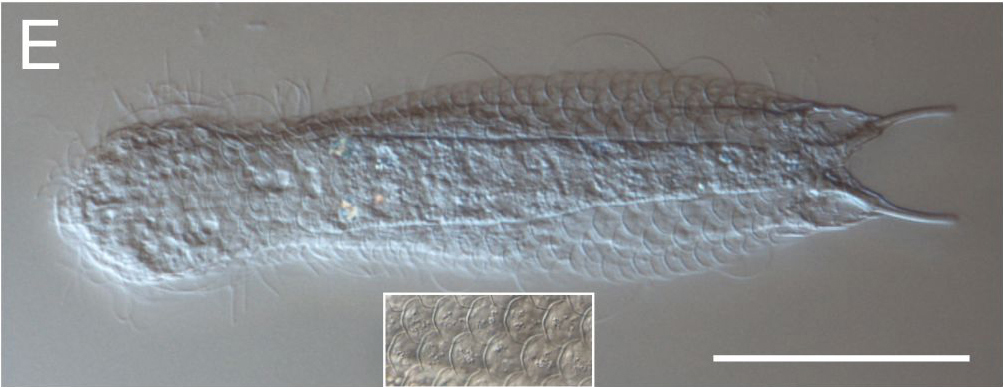

Supplement: Supplementary material 6 — Figure 1E: Fam. Chaetonotidae, Lepidodermella squamata (insert shows the dorsal scales); Scale bar =100 µm. [file biodiversity_data_journal-3-e5800-s006.jpg]

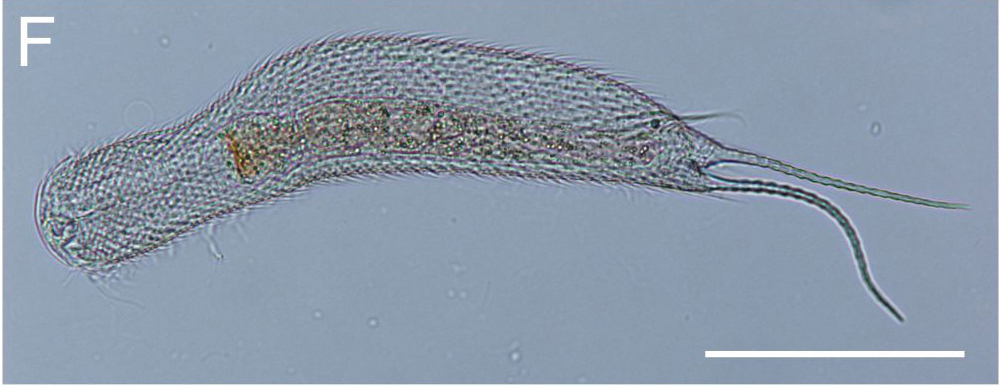

Supplement: Supplementary material 7 — Figure 1F: Fam. Chaetonotidae, Polymerurus nodicaudus; Scale bar =100 µm. [file biodiversity_data_journal-3-e5800-s007.jpg]

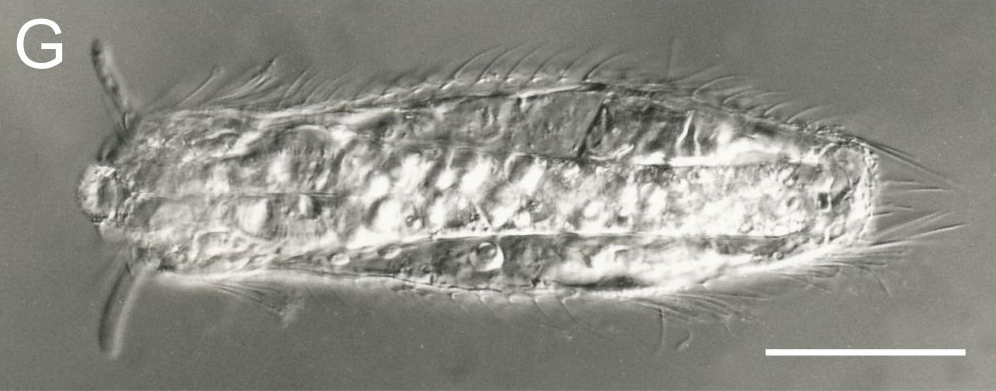

Supplement: Supplementary material 8 — Figure 1G: Fam. Neogosseidae, Neogossea antennigera; Scale bar = 50 µm. [file biodiversity_data_journal-3-e5800-s008.jpg]

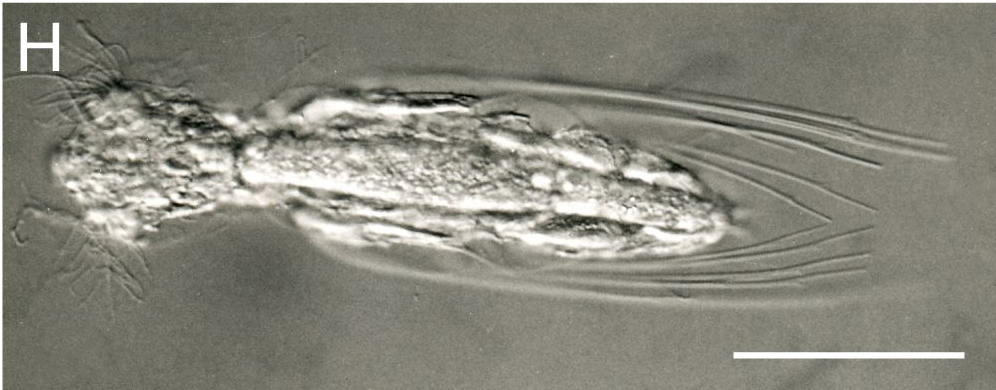

Supplement: Supplementary material 9 — Figure 1H: Fam. Dasydytidae, Stylochaeta fusiformis; Scale bar = 50 µm. [file biodiversity_data_journal-3-e5800-s009.jpg]
